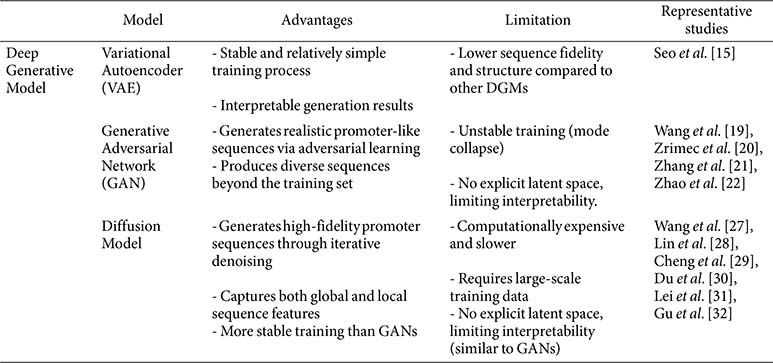# Deep Generative Model–Driven Design of Microbial Synthetic Promoters

**DOI:** 10.4014/jmb.2510.10043

**Published:** 2025-11-26

**Authors:** Euijin Seo, Doeon Sung, Jeong Wook Lee

**Affiliations:** 1 Department of Chemical Engineering, Pohang University of Science and Technology (POSTECH), Pohang 37673, Republic of Korea; 2 School of Interdisciplinary Bioscience and Bioengineering, Pohang University of Science and Technology (POSTECH), Pohang 37673, Republic of Korea

**Keywords:** Synthetic promoter, deep learning, deep generative model, differential RNA sequencing

## Abstract

A synthetic promoter is an artificially designed DNA sequence based on naturally occurring promoter elements, enabling more precise control of gene expression than natural promoters. Design of synthetic promoters with tunable expression levels is key to precise genetic regulation in microbes, supporting metabolic engineering, natural product biosynthesis, and diverse biotechnological applications. Recent advances in deep learning have made it possible to generate functional synthetic promoters using deep generative models (DGMs). Such approaches dramatically accelerate the traditionally labor-intensive and time-consuming process of experimental promoter design, enabling the efficient discovery of synthetic promoters. In synthetic promoter generation, three major types of DGMs have been predominantly employed: variational autoencoders (VAEs), generative adversarial networks (GANs), and diffusion models. VAEs reconstruct promoters through latent feature learning, GANs create realistic promoter sequences via adversarial training, and diffusion models iteratively denoise random inputs to generate high-fidelity synthetic promoters. This review outlines deep learning–based strategies for synthetic promoter design, encompassing data acquisition, promoter generation, and validation of promoters generated by DGMs.

## Introduction

Promoters are key gene sequences on DNA that regulate the initiation of transcription. They possess unique structural features that distinguish them from other DNA sequences, enabling RNA polymerase (RNAP) to bind, unwind the DNA, and initiate messenger RNA (mRNA) synthesis [1]. Promoters are typically located upstream of the transcription start site (TSS), and their sequences and lengths vary depending on the strain or gene. The composition of the promoter sequence affects its binding affinity with RNAP and the strength of interactions with transcription factors, which directly influences the promoter's expression level [2, 3].

Within microorganisms, diverse genes are expressed. Some genes are expressed strongly, while others are expressed weakly. Regulating this expression strength plays a crucial role in strain growth and improvement. Since gene expression levels are determined by the sequence structure of the promoter [2], securing diverse promoter libraries for each strain becomes a key means to precisely control gene expression. By securing promoters of varying strengths, enzyme expression can be finely tuned to optimize metabolic pathways [4-11]. Furthermore, adjusting the sensitivity of transcription factor expression can significantly enhance the performance of existing genetic circuits [12-14]. For these reasons, many researchers have actively pursued studies to construct promoter libraries from diverse microorganisms.

Advances in microbiology and molecular biology have increased the need for sophisticated control over more genes within strains. Consequently, building promoter libraries with a broader range of expression intensities than those found in nature has become increasingly important. Consequently, research on 'synthetic promoters'—artificially designed promoters not found in nature—is actively underway.

Deep learning technologies are being actively utilized in various synthetic biology studies. Deep learning enables analysis, prediction, and generation of new data by learning complex characteristics within data. Leveraging these advantages, synthetic biology has applied deep learning to discover new patterns in experimental data and generate biological sequences. In particular, deep generative models (DGMs) such as Variational Autoencoders (VAE) [15-18], Generative Adversarial Networks (GAN) [19-26], and diffusion models [27-33] are being utilized as important tools for generating biological data. These approaches are widely used to design synthetic promoters. DGMs can learn complex promoter patterns to generate functional yet novel sequences. This approach enables rapid and efficient design of ‘functional’ promoters with various expression levels compared to traditional experimental methods.

**Fig. 1:**
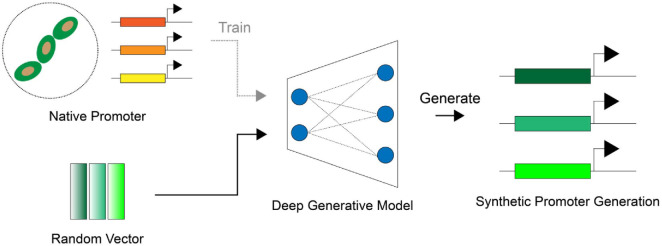
Overall workflow for synthetic promoter generation using DGMs. In the generation phase, a DGM is trained on native promoter sequences to learn their structural and compositional characteristics. Once trained, the DGM generates synthetic promoters by sampling random latent vectors, producing novel sequences that reflect the learned promoter features.

This paper presents an overview of synthetic promoter design using DGMs (Fig. 1). It systematically discusses: (1) approaches for obtaining training datasets in synthetic promoter studies, (2) strategies for generating and validating synthetic promoters using DGMs such as VAEs, GANs, and diffusion models, and (3) methodologies for assessing the quality of DGM-generated promoters.

## Acquisition of Training Data Libraries for Synthetic Promoters

To train deep learning models for synthetic promoter generation, large and reliable promoter sequence datasets are essential. Initial efforts primarily relied on information compiled from literature and public databases. With the advent of RNA-sequencing–based approaches, it became possible to estimate promoter activity on a large scale and with quantitative accuracy.

### Acquisition of Existing Database-Based Promoter Libraries

Promoters are key biological components regulating gene expression, and their sequences have been studied across various species. As research accumulated, databases systematically organizing promoter information for each species were established. Representative examples include RegulonDB [34] specialized for *E. coli*, DBTBS [35] for *B. subtilis*, and YeasTSS [36] for *S. cerevisiae*. Numerous studies have utilized existing promoter databases to construct training datasets for DGMs. For instance, Zrimec *et al*. [20] built such a dataset by extracting regulatory sequences containing promoters of *S. cerevisiae* S288C from the Saccharomyces Genome Database [37].

### Acquisition of Promoter Libraries through Computational Prediction Approaches

For major model strains such as *E. coli* or *S. cerevisiae*, a wide range of promoter sequences and their corresponding expression data are already available. However, for less-studied non-model strains, or even for model strains where promoter activity under specific environmental conditions is of interest, obtaining the promoter sequences of the target strain becomes an essential step.

To address this challenge, various computational tools have been developed to detect promoter candidates from whole-genome sequences. For example, BPROM [38] scans the input sequence to detect candidate sequences presumed to be the −35 box and −10 box, and predicts the promoter location based on these. Beyond this traditional motif-based sequence analysis approach for identifying promoters, prediction models based on machine learning and deep learning are also being actively developed [39-42].

Indeed, Zhao *et al*. performed RNA-sequencing (RNA-seq) on *Halomonas* DT01, a strain with relatively scarce research, and utilized BPROM to predict promoter locations based on the −10 and −35 motifs, thereby constructing a promoter dataset for this strain [22].

### Acquisition of a Promoter Library Based on Differential RNA-Sequencing

Although computational tools can predict promoter locations in a strain, promoter sequences do not necessarily contain −10 and −35 motifs, and the sequence structures between promoters vary significantly. Therefore, a method is needed to experimentally confirm the promoter locations in the strain.

Bischler *et al*. were the first to identify *Helicobacter pylori*'s TSS using differential RNA-seq (dRNA-seq) [43]. Unlike existing computational tools, dRNA-seq is a technique that experimentally determines the TSS of a strain. This method is performed by combining traditional RNA-seq with terminator exonuclease (TEX) treatment.

Intracellular RNA is broadly categorized into primary transcripts, transcribed directly from the TSS, and processed RNA, which undergoes subsequent biological cleavage. Primary transcripts possess a triphosphate structure at their 5' end, whereas processed RNA has a monophosphate structure. TEX recognizes and degrades RNA at these monophosphate ends. Consequently, in TEX-treated samples, processed RNA is removed, leaving only primary transcripts selectively intact.

**Fig. 2:**
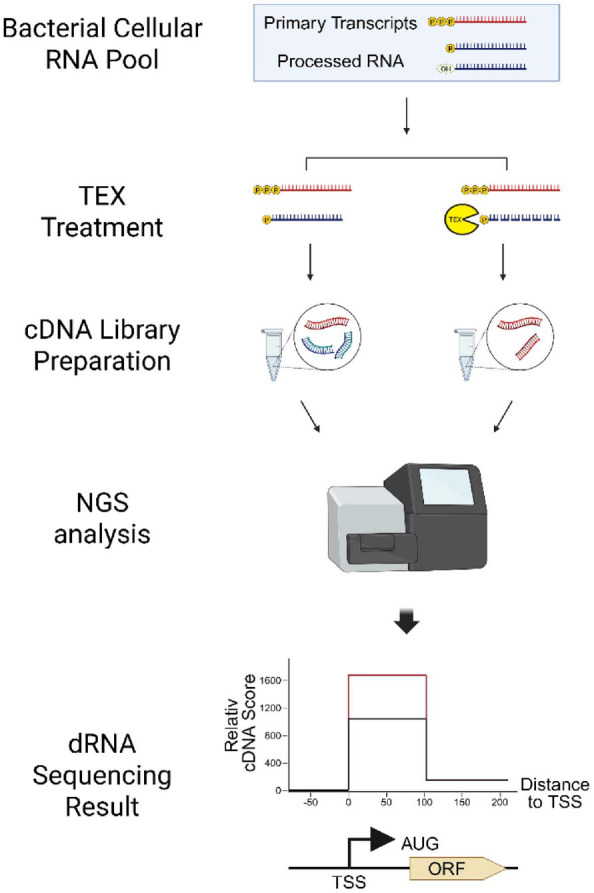
Overview of dRNA-seq for genome-wide TSS identification. In dRNA-seq, the total RNA pool is divided into a TEX-treated (+TEX) and an untreated (−TEX) sample. Each RNA sample is reverse-transcribed into cDNA for RNA-seq analysis. The resulting read distributions are compared using TSSpredator, a dedicated dRNA-seq analysis tool, to identify TSSs. Regions showing distinct enrichment exclusively in the TEX-treated sample indicate the positions of primary transcripts. By determining the TSSs of a given strain, the downstream sequences can be defined as promoter regions, and their expression levels can subsequently be quantified.

In dRNA-seq, the total RNA pool is divided into a TEX-treated group (+TEX) and an untreated group (−TEX). Each sample’s RNA is reverse transcribed into cDNA for RNA-seq analysis. Subsequently, the distribution of the obtained reads is compared using TSSpredator [44], a dedicated dRNA-seq data analysis tool, to predict the TSS of each transcript. As a result, locations showing distinct enrichment appear only in the TEX-treated group. This pattern enables genome-wide identification of the starting point of the primary transcript, i.e., the TSS location. By identifying the TSS of a strain, the upstream region of the TSS can be predicted as the promoter sequence, and its expression level can be confirmed (Fig. 2).

Starting with the work of Bischler *et al*., dRNA-seq has been performed on various bacterial strains [45, 46]. The data obtained from these studies provided promoter sequences and expression levels, serving as foundational data for deep learning analyses in subsequent research. Notably, Thomason *et al*. [45] performed genome-wide dRNA-seq on *E. coli* K12 MG1655 to systematically identify TSSs, which subsequently enabled numerous deep learning–based promoter studies [19, 27, 28, 30, 31]. Additionally, Kopf *et al*. [46] performed dRNA-seq on the cyanobacterial strain *Synechocystis* sp. PCC6803. Subsequently, Seo *et al*. [15] utilized this data to build a deep learning model for generation of cyanobacterial promoter sequences.

## Design of Synthetic Promoters Using DGMs

DGMs can identify features in training data and generate novel data that resembles existing data while being previously unseen. Conventional synthetic promoters were created by combining different promoter sequences [47-52] or introducing mutations via error-prone PCR [7, 54-56]. However, these methods not only limit the number of sequences that can be generated but also offer no guarantee that the resulting promoters will actually function. Above all, they rely on experimental trial and error, consuming significant time and effort.

In contrast, DGMs enable the model itself to learn the characteristics of promoter sequences and efficiently generate new sequences similar to natural promoters based on this knowledge. Thanks to these advantages, DGMs are being actively adopted for promoter design in the synthetic biology field. Initially, models like VAE or GAN were utilized, and recently, diffusion models—known for their excellent data generation capabilities—are also being actively applied to synthetic promoter generation research. The overall advantages and limitations of various DGM architectures are summarized in Table 1.

### Variational Autoencoder (VAE)

VAE [57] was proposed alongside GANs as a representative model in early DGM research. VAE evolved from the Autoencoder (AE) structure [58], designed to efficiently represent sequence data as numerical vectors. Computers excel at processing numerical data but struggle to directly understand non-numeric data like words (*e.g.*, sunshine, puppy, telephone), DNA sequences (*e.g.*, TTGACATT), or images. For example, a synthetic biologist recognizes TTGACATT as a DNA sequence, but a computer perceives it as a simple collection of characters (ASCII codes), not the biological meaning of the nucleotide sequence. In deep learning, such data must be converted into numeric vectors for the model to process. However, it is not sufficient to merely convert it into vectors; a process is needed to represent data that is semantically similar as being close together in the vector space, and data that is not similar as being far apart. This process is called data embedding, and through this, a mathematical representation space—the latent space—is formed where semantically similar data points are clustered in close proximity.

**Fig. 3:**
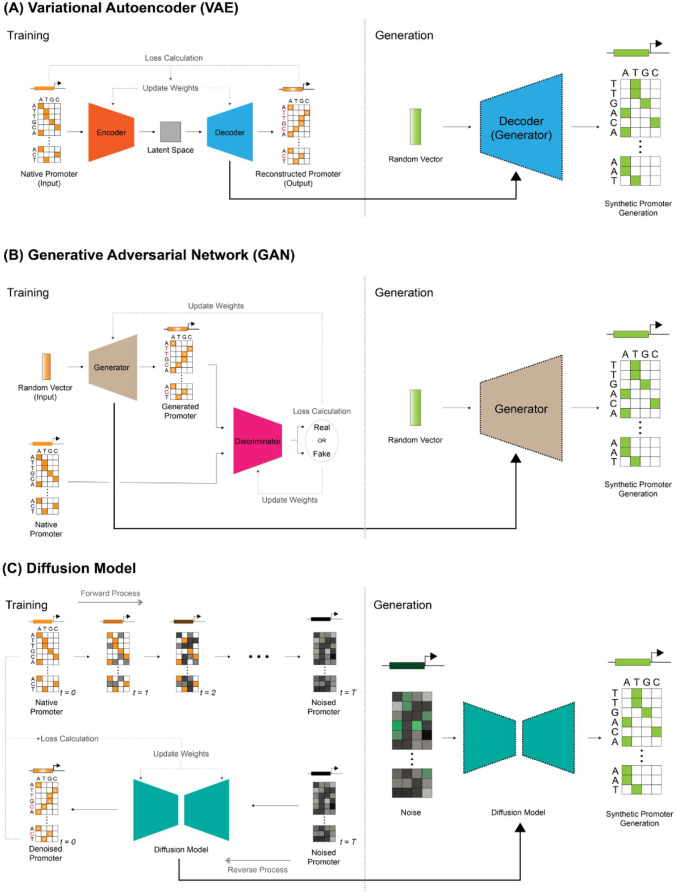
Synthetic promoter sequence generation using DGMs. (**A**) VAE-based promoter generation. A VAE is trained on natural promoter sequences to capture their latent features. The encoder embeds input sequences into a latent space, while the decoder reconstructs realistic promoter sequences from these latent representations. After training, random vectors sampled from the latent space can be decoded into novel synthetic promoters that reflect the learned promoter characteristics. (**B**) GAN-based promoter generation. A GAN consists of a generator that attempts to produce realistic promoter sequences and a discriminator that distinguishes real promoters from generated ones. Through adversarial training, the generator progressively learns to produce promoter-like sequences, while the discriminator becomes better at detecting synthetic ones. Once trained, the generator can create synthetic promoters from random input vectors. (**C**) Diffusion model–based promoter generation. In diffusion models, training involves two processes: a *forward process* that gradually adds noise to real promoter sequences until the data are completely randomized, and a *reverse process* in which the model learns to denoise and reconstruct the original promoter sequences. After training, the diffusion model can generate realistic synthetic promoters by transforming random noise vectors into structured DNA sequences that capture natural promoter features.

VAE is a DGM that modifies the existing AE structure for data generation purposes. It is designed not only to embed data but also to directly generate new data. VAE consists of two main components: an encoder and a decoder. First, the encoder embeds the characteristics of the input sequence data into the latent space as a vector through operations with internal weights. This latent space acts as a sequence map, numerically representing the functional and structural similarities between sequences. Subsequently, the decoder reconstructs data of a size similar to the input sequence based on the latent vector generated by the encoder. The model calculates the difference between the input sequence and the reconstructed sequence to determine the loss, then iteratively adjusts the weights of the encoder and decoder to minimize this loss. Repeating this process across numerous sequence datasets enables the encoder to gradually form a sophisticated latent space reflecting the semantic features of sequences, while the decoder becomes capable of generating new sequences within this space that resemble actual promoters. After training is complete, by inputting a random vector resembling the learned sequence vectors within the latent space into the decoder, new synthetic promoter sequences exhibiting the characteristics of real promoters can be generated (Fig. 3A).

VAE offers relatively stable and fast model training, and its structure is simpler compared to other DGMs. Furthermore, analyzing the latent space allows observation of how sequence similarity manifests in the data [59]. However, the quality of generated data is relatively lower compared to other DGMs, increasing the likelihood of producing sequences with less distinct structural features than other generative models [60].

VAE has been utilized in various data generation studies and has also been applied to synthetic promoter design. Seo *et al*. [15] trained a VAE on dRNA-seq data from *Synechocystis* sp. PCC 6803 to learn latent representations of cyanobacterial promoter features. During training, the encoder and decoder were jointly optimized by minimizing the reconstruction loss between native and reconstructed promoters, enabling the model to learn latent representations that captured key promoter features. In the generation phase, the decoder alone was used to create approximately 10,000 synthetic promoters from random latent vectors. After evaluation of the synthetic promoters, they showed that the generated sequences preserved key promoter motifs while exhibiting distinct sequence diversity.

### Generative Adversarial Network (GAN)

GANs [61] were the most widely used representative DGMs until the emergence of diffusion models. A GAN consists of two neural networks: a Generator and a Discriminator. The Generator takes a random vector as input and generates a fake promoter sequence through weighted operations. Conversely, the Discriminator takes both real promoter sequence data and the fake sequences generated by the Generator as input, distinguishing whether each data point is ‘real’ or ‘fake’. This classification result is reflected in the training of both networks: the Generator adjusts its weights to generate sequences that are more realistic and can deceive the Discriminator, while the Discriminator adjusts its weights to distinguish between fake and real sequences more accurately. Through repeated rounds of this adversarial training, both networks push each other’s performance to higher levels until they reach an equilibrium state. Once training is complete, feeding the Generator a simple random vector allows it to generate new promoter sequences that closely resemble real data (Fig. 3B).

Following the development of VAEs, GANs emerged as another major class of DGMs, characterized by an adversarial training mechanism between a generator and a discriminator. Compared to VAEs, GANs can generate higher-quality data and effectively learn complex data distributions and patterns [60]. However, despite their impressive ability to generate high-quality data, GANs also have inherent limitations. If the learning balance between the Generator and Discriminator is not properly maintained, the Generator may repeatedly produce only a limited form of data. This phenomenon, known as mode collapse, is considered a major challenge in GAN training [62]. Furthermore, while VAEs allow for relatively interpretable insights into the semantic structure of data through the latent space, GANs have limitations in explicitly controlling the generation process or interpreting what characteristics the model has learned [60]. Nevertheless, GANs remain widely used in data generation due to their strength in producing sharp, high-fidelity data. Leveraging this characteristic, synthetic biology has actively adopted GANs as models for generating high-quality synthetic promoter sequences.

Wang *et al*. were the first to apply GANs to promoter design [19], training WGAN-GP on *E. coli* data and confirming that the generated sequences faithfully reflected the motifs and structural characteristics of native promoters. In this study, the model was trained to enable the generator of the GAN to produce sequences that more closely resembled *E. coli* native promoters, while the discriminator was optimized to distinguish native from synthetic promoters with higher precision. After training, high-quality *E. coli* synthetic promoters were generated by feeding random latent vectors into the generator. Wang *et al*. addressed training instability and mode collapse issues inherent in GANs by introducing the ‘Wasserstein GAN with Gradient Penalty (WGAN-GP)’, a GAN model variant that enhances training stability, and demonstrated superior performance over other GAN-based models.

Subsequent studies have explored various applications. Zhang *et al*. [21] combined GANs with a genetic algorithm (GA), an evolution-inspired optimization method, to design inducible promoters preserving the LacI binding site. Zhao *et al*. [22] applied GANs to design *Halomonas* TD01 promoters adaptable to high-salinity environments. Zhou *et al*. [23] integrated GANs with reinforcement learning (RL), a strategy for goal-directed optimization, to generate *E. coli* BL21 promoters exhibiting desired expression levels.

### Diffusion Model

The diffusion model [63], alongside GPT, is one of the most prominent generative models recently gaining significant attention. It is highly regarded for its ability to generate data that is far more sophisticated and clear compared to existing models. Inspired by the ‘diffusion’ phenomenon, the diffusion model derives its name from the physical process in which particles gradually spread from regions of high concentration to low concentration. Analogously, the model progressively adds noise that “diffuses” through the data in the forward process and then learns to reverse this corruption to recover the original distribution.

The learning process of the diffusion model consists of two main stages: the forward process and the reverse process. First, in the forward process, the actual promoter sequence data is progressively transformed (“noised”) over multiple steps. This process involves repeatedly adding a vector composed of random noise to the input data. Performing this operation over multiple steps gradually causes the structural features of the original data to be lost. After a sufficient number of steps, the promoter sequence data no longer retains its original information and transforms into a state close to pure noise.

Subsequently, the reverse process performs the inverse operation, conducting denoising to restore the original data form from the noise. While the forward process can be expressed as simple probabilistic noise addition, the reverse process is difficult to define mathematically with an explicit inverse function. Therefore, deep learning models are designed to learn how to remove noise at each step. This enables the model to take random noise as input and progressively generate high-quality synthetic data (Fig. 3C).

Diffusion models can generate new data that is highly realistic and diverse, going beyond merely reproducing training data. Particularly due to their advantages of stable training and high fidelity, they are now widely used for generating various biological data, such as protein structures [64], protein–ligand interactions [65], and DNA sequences [33]. Although, like GANs, it is difficult to interpret which sequence features the model used to generate synthetic promoters, and it requires high computational resources due to its greater computational load compared to other DGMs. Nevertheless, thanks to its training stability, data diversity, and excellent generation quality, the diffusion model has become the most dominant DGM currently in use.

Wang *et al*. [27] were the first to employ a diffusion model to design synthetic promoter sequences in *E. coli* BL21.They gradually added noise to native promoters, thereby corrupting their original sequence features, and utilized a U-Net architecture—a convolutional neural network known for its symmetric encoder–decoder structure and strong feature-preserving capability—to iteratively denoise the data and reconstruct promoter-like sequences from the noised inputs. Subsequently, the researchers generated high-quality *E. coli* synthetic promoters by feeding randomly noised latent vectors into the trained U-Net.

Since then, numerous studies have further explored promoter design based on diffusion models. Lin *et al*. [28] demonstrated that diffusion models can generate more stable synthetic promoter sequences compared to GANs, using data from *E. coli* MG1655 and *S. cerevisiae*. Furthermore, Du *et al*. [30] applied the Multinomial Diffusion Model (MDM) to obtain synthetic sequences of higher quality than existing diffusion models. Lei *et al*. [31] reported that sequences generated from *E. coli* K12 MG1655 and *Synechocystis* sp. PCC6803 exhibited superior characteristics compared to those from VAE-based models.

## Sequence-Based Quality Assessment of DGM-Generated Synthetic Promoters

DGMs use unsupervised learning, allowing model training without predefined sequence features and capturing patterns beyond human-designed rules. However, its interpretability and performance evaluation remain challenging, requiring additional analyses to confirm biological relevance. In synthetic promoter generation, this involves comparing sequence characteristics—such as k-mer frequency and motif patterns—between generated and natural promoters. [15, 19-23, 27-32].

Another key goal of DGM-based sequence generation is to ensure diversity, producing promoters that not only reflect natural characteristics but also differ from existing sequences. Accordingly, many studies use BLAST [66], to evaluate the similarity and distinctiveness between synthetic and real promoters.

### k-mer Frequency Analysis

k-mer frequency analysis is a widely used method for verifying the quality of synthetic promoters. A k-mer refers to a contiguous sequence of bases of length k. For example, when k = 4, this corresponds to 4-bp fragments like 'ATGC' or 'TAAT'. This analysis calculates the occurrence frequency of all k-mers in both synthetic promoter data and actual promoter data, then compares the two distributions.

Since key motifs like the Pribnow box in promoters are typically 6 bp in length, the frequency of 2-mers to 6-mers is usually set as the primary analysis target. The k-mer distributions of synthetic and real promoters can be quantitatively compared using statistical metrics like the Pearson correlation coefficient. If the two distributions are similar, the model can be evaluated as having well-learned the sequence characteristics of real promoters. Furthermore, by examining the k-mer frequencies of well-conserved motifs like the −10 box, one can indirectly infer the likelihood that the generated sequence set could function as a real promoter.

Thus, k-mer frequency analysis provides a simple yet intuitive method for verifying the generation quality of synthetic promoters and is widely used as a quality assessment metric in most DGM-based promoter generation studies [15, 19, 21-23, 28-32]. This analysis has been applied not only to direct comparisons of overall k-mer distributions but also to evaluating the frequency of key promoter motifs, such as the Pribnow box [19], at specific sequence positions.

Many studies have employed k-mer frequency analysis to evaluate the sequence fidelity of synthetic promoters generated by DGMs. Wang *et al*. [19] compared several deep-learning-based promoter generators using the Pearson correlation of 6-mer frequencies between generated and training data, showing that WGAN-GP achieved the highest sequence realism. Seo *et al*. [15] analyzed the positional distribution of 6-mers—such as *TATAAT*, *TAAAAT*, *TAGAAT*, and *AAAATA*, which are frequently observed in native cyanobacterial promoters—and found that VAE-generated promoters reproduced native-like positional patterns. Similarly, multiple studies have confirmed through k-mer frequency analyses that diffusion- and GAN-based models (*e.g.*, Diffusion, PromoterDiff, MDM, DDPM, and PromoDGDE) effectively captured the native sequence characteristics of *E. coli*, *S. cerevisiae* promoters [21-23, 28-32].

### Sequence Motif Analysis

Sequence motif analysis is a method for comparing whether motifs observed within synthetic promoter sequences are similar to motifs in actual promoters. To do this, the frequency of base occurrence at each position is calculated from the synthetic promoter data, and a positional weight matrix is derived based on this. The results of the positional weight matrix can be visualized using tools like sequence logos [67]. This allows us to identify which sequences are conserved and which motifs appear in the synthetic promoter data. Although different promoter motifs exist across various strains, the most prominently conserved element in sequence logo analysis is the −10 box, and the −35 box is also observed to be conserved. By confirming that synthetic promoters and actual promoters share common motifs, we can validate that DGMs generate synthetic sequences reflecting the core motifs of actual promoter sequences.

Sequence motif analysis is the most intuitive approach for verifying results among various synthetic promoter quality validation methods and has been widely used for quality assessment in many DGM-based synthetic promoter generation studies [15, 19, 28-31].

### BLAST Analysis

The quality analysis methods mentioned earlier focus on evaluating how well the synthetic promoter reflects the characteristics of the actual promoter. However, another purpose of sequence generation using DGMs is to obtain diverse sequences, so possessing distinctiveness and diversity that differentiates them from existing sequences is also important.

To demonstrate this diversity, many studies have utilized BLAST (Basic Local Alignment Search Tool) [66], a widely used sequence analysis tool. BLAST is a tool that rapidly searches for local similarity by comparing a given sequence to sequences in a database, producing an E-value as a result. The E-value represents the expected number of times an alignment of the same level would occur by chance in a random search; a lower value indicates that the sequence has statistically significant similarity to sequences in the database.

Several research teams compared synthetic promoter sequences to actual bacterial promoter sequences using BLAST and analyzed the resulting E-value distributions. Synthetic promoters generated by DGMs exhibited a lower frequency of E-value distribution compared to randomly combined sequences (random promoters). This demonstrates that DGMs generate diverse sequences while better reflecting the characteristics of actual promoters than simple random sequences [15, 19, 21, 29, 30, 32].

Wang *et al*. [19] reported that GAN-generated *E. coli* sequences were structurally distinct from the native genome. Similarly, Seo *et al*. [15] found that VAE-generated promoters in *Synechocystis* sp. PCC6803 showed low sequence similarity to native promoters. Cheng *et al*. [29], Du *et al*. [30], and Gu *et al*. [32] also confirmed through BLAST analyses that synthetic promoters generated by models such as PromoterDiff, Multinomial Diffusion, and PromoDGDE exhibited sequence patterns markedly different from those in native *E. coli* and *S. cerevisiae* genomes.

## Conclusion

This study focused on DGMs for synthetic promoter design, highlighting representative architectures such as VAEs, GANs, and diffusion models. These DGMs enable efficient generation of promoter sequences with diverse and tunable expression levels, thereby facilitating precise control of gene expression in microbial systems. Recent advances extend this approach to large language models [68, 69] and integrated toolkits that automate generation and prediction within a single pipeline [70]. In this way, more advanced and sophisticated deep learning models continue to be employed in synthetic promoter generation research. By leveraging data-driven generation rather than manual sequence engineering, generative models provide a powerful framework for expanding promoter diversity and accelerating strain optimization across environmental, industrial, and therapeutic applications.

This paper presented a deep learning–based framework for synthetic promoter generation and expression prediction, summarizing key promoter features, data acquisition methods, and representative DGMs such as VAE, GAN, and diffusion models. Along with high-throughput techniques like MPRA, these developments are accelerating the creation of large, high-quality promoter libraries, driving more precise and efficient design in synthetic biology.

The DGM–based framework for synthetic promoter design can also be applied to non-model but industrially valuable microorganisms. Unlike *E. coli* and other well-studied model organisms, these strains have received limited research attention despite their unique physiological or metabolic advantages—such as high tolerance to extreme environments or efficient metabolite production—which make them attractive for industrial biotechnology. However, the lack of robust genetic tools and comprehensive omics data makes their experimental manipulation and fine-tuning of gene expression challenging.

Applying DGMs to non-model species further involves difficulties such as limited high-quality datasets, inconsistent promoter annotation, and uncertainty in capturing species-specific regulatory architectures. Nevertheless, DGMs possess key advantages, including the ability to learn sequence features from limited data and generate functional promoters without extensive prior biological knowledge. Owing to these strengths, DGM-based studies on synthetic promoters for non-model strains have begun to emerge. For instance, Zhao *et al*.[22] applied a DGM-based approach using dRNA-seq data from the halophilic bacterium *Halomonas* TD01 to generate synthetic promoters, illustrating the potential of DGMs to extend promoter design beyond model organisms. However, this remains an early demonstration, and further progress in data quality, model generalization, and experimental validation will be essential for broader application across diverse non-model systems.

Ultimately, deep learning–based synthetic promoter design efficiently generates sequences with diverse expression levels, paving the way for precise tuning of gene expression in microbial strains. This approach is expected to significantly enhance the productivity and efficiency of microbial strains across various fields, including environmental, industrial, and therapeutic applications.

## Figures and Tables

**Table 1:** Comparison of DGMs for synthetic promoter generation.